# Blueprint2Code: a multi-agent pipeline for reliable code generation via blueprint planning and repair

**DOI:** 10.3389/frai.2025.1660912

**Published:** 2025-10-17

**Authors:** Kehao Mao, Baokun Hu, Ruixin Lin, Zewen Li, Guanyu Lu, Zhengyu Zhang

**Affiliations:** School of Information Science and Technology, Hangzhou Normal University, Hangzhou, China

**Keywords:** code generation, large language models, multi-agent systems, program synthesis, automated debugging, blueprint planning

## Abstract

Automated programming has become a powerful tool for solving real-world problems. Code generation, in particular, plays a key role in improving developer productivity and reducing the entry barrier to software development. Recent advances in large language models (LLMs) have significantly improved program synthesis, enabling high-quality code generation from natural language. However, LLMs still struggle with complex tasks, especially in understanding problem intent, conducting multi-step reasoning, and producing code that passes all test cases. As task difficulty increases, existing models often fail to devise complete and reliable generation strategies, leading to reduced accuracy and robustness. To address these limitations, we propose Blueprint2Code, an innovative multi-agent framework for code generation. It emulates the human programming workflow through the coordinated interaction of four agents—Previewing, Blueprint, Coding, and Debugging—forming a closed-loop system from task comprehension to planning, implementation, and iterative refinement. Compared to existing methods, Blueprint2Code shows superior performance on complex programming tasks. Extensive experiments on benchmark datasets—HumanEval, MBPP, their extended versions (HumanEval-ET, MBPP-ET), and the APPS competition dataset—demonstrated its effectiveness, achieving strong pass@1 results: HumanEval 96.3%, MBPP 88.4%, HumanEval-ET 86.5%, MBPP-ET 59.4%, and APPS 24.6%. The related code is available at https://github.com/MKH99918/Blueprint2Code.

## 1 Introduction

Automated code generation is a pivotal area within computer science, aiming to reduce human intervention by automatically producing computer code and demonstrating significant practical value in various real-world scenarios (Barone and Sennrich, 2017; Li et al., 2022; Parvez et al., 2018). With the rapid development of large language models (LLMs) (Naveed et al., 2023; Chang et al., 2024; Rillig et al., 2023), researchers have achieved remarkable progress in code generation. However, existing methodologies still face substantial limitations when confronted with complex programming tasks, particularly in terms of comprehending problem requirements, performing multi-step reasoning, and generating function code capable of passing complete test suites (Islam et al., 2023, 2022).

Although LLMs have shown formidable capabilities in code generation (Naveed et al., 2023; Chang et al., 2024; Rillig et al., 2023), especially in function completion and simple task generation, notable deficiencies persist when dealing with complex tasks. Currently, mainstream approaches can be broadly classified into two categories: The first category encompasses prompt-based direct generation methods, such as Direct, Chain-of-Thought (CoT), and Self-Planning (Wei et al., 2022; Jiang et al., 2024), which generate code through static reasoning but often lack global planning and controllability. The second category introduces self-feedback mechanisms, such as Reflexion and MapCoder (Islam et al., 2024; Shinn et al., 2023), which enhance generation quality by guiding models to self-check or self-plan; however, these processes predominantly rely on closed-loop reasoning within a single model, lacking explicit modular structures and interstage collaboration, thus struggling to simulate the complete process of “preview-planning-coding-debugging” in real-world development workflows.

This limitation is particularly pronounced in programming competition datasets like APPS (Hendrycks et al., 2021), where tasks impose stringent requirements on input-output formats and involve complex algorithms, multi-step logic, boundary handling, and performance optimization, serving as critical test scenarios for evaluating the comprehensive capabilities of code generation systems. Existing models frequently fail to generate code that passes all test cases in a single attempt, exposing gaps in context retention, task decomposition, and long-range dependency modeling (Munkhdalai et al., 2024; Fountas et al., 2025). Therefore, constructing a code generation system with clearly defined stages, information flow coordination, and error repair capabilities remains an urgent challenge.

**Figure 1:**
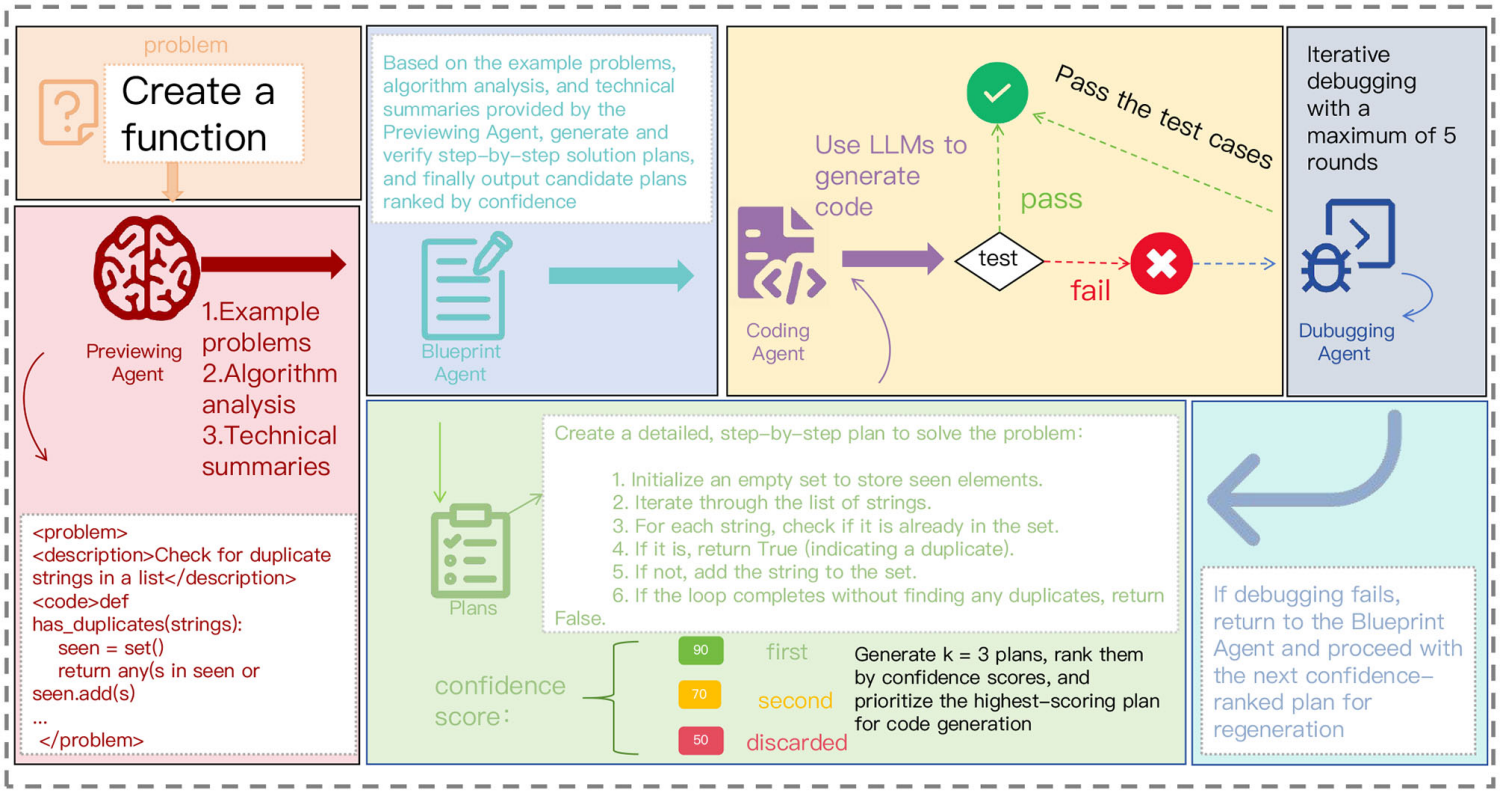
Overview of Blueprint2Code. The pipeline begins with a Preview Agent that learns relevant algorithms and techniques, followed by the generation of a detailed blueprint plan (including technical summaries and planning examples). It then proceeds to the Coding and Debugging Agents.

To address these challenges, we propose Blueprint2Code, a multi-agent collaborative framework designed for complex code generation tasks. This framework draws inspiration from the workflow of human programmers and incorporates four key stages: task preview, blueprint planning, code implementation, and debugging optimization, each executed by dedicated agents that collaborate dynamically through a unified control strategy. Compared to end-to-end methods, Blueprint2Code offers enhanced modular controllability and stage interpretability, significantly improving the model's capabilities in task decomposition, structural modeling, and error repair. Meanwhile, this method demonstrates excellent transferability, achieving significant improvements not only in high-performance models such as GPT-4o and GPT-3.5-turbo but also exhibiting stable advantages in small-scale models such as GPT-4o-mini. This indicates broader application potential and deployment adaptability. Figure 1 provides an overview of the Blueprint2Code workflow.

We conducted extensive experiments on two popular benchmark datasets, HumanEval and MBPP (Chen et al., 2021; Austin et al., 2021), along with their extended test sets, HumanEval-ET and MBPP-ET, as well as the public programming competition dataset APPS (Hendrycks et al., 2021). By utilizing ChatGPT and GPT-4 (Achiam et al., 2023), we demonstrated that our method significantly enhances the problem-solving capabilities of large language models (LLMs), outperforming current mainstream approaches such as Chain-of-Thought (CoT) and MapCoder (Islam et al., 2024). Furthermore, in the field of code generation, although large language models have showcased their formidable capabilities, the practicality of smaller models remains crucial in many real-world applications, particularly in resource-constrained environments. On the 8B-parameter GPT-4o-mini model, our method, Blueprint2Code, still proved effective, achieving a result of 89.1% on the HumanEval dataset, surpassing MapCoder's 88.4%, CoT's 87.2%, and the 84.7% achieved by direct code generation. This provides strong support for its widespread promotion in future applications involving small models.

The primary contributions of this paper include:

- We propose an innovative multi-agent code generation framework, Blueprint2Code, which simulates the “previewing-planning-coding-debugging” programming process of human programmers and elaborately designs the collaboration mechanism among four types of agents.
- We conducted extensive experiments on HumanEval, MBPP, their extended test sets HumanEval-ET and MBPP-ET, as well as the public programming competition dataset APPS, systematically evaluating the performance of Blueprint2Code in terms of code generation quality, pass rate, and adaptability to small models.
- The experimental results demonstrate that Blueprint2Code significantly outperforms existing methods (such as CoT, Reflexion, MapCoder, etc.) on mainstream evaluation benchmarks and maintains excellent performance in resource-constrained small model environments, showcasing its strong versatility and practical deployment potential.

## 2 Materials and methods

### 2.1 Relate work

#### 2.1.1 Prompt engineering

With the widespread application of large language models (LLMs) in code generation tasks, prompt engineering has been recognized as one of the key approaches to enhancing model generalization capabilities and controllability (White et al., 2023; Giray, 2023). In recent years, numerous studies have focused on designing more instructive prompt structures to guide models in conducting self-feedback, self-correction, and logical reasoning during the generation process. For instance, the self-refinement method improves semantic accuracy and structural integrity by providing high-quality examples and recursive calls, enabling the model to iteratively refine its own code output over consecutive rounds. Meanwhile, the self-debugging strategy compares model outputs with existing test cases and automatically generates repair suggestions, even enabling error localization through natural language explanations of the code in the absence of test data. Additionally, some approaches, such as CodeCoT, introduce a chain-of-thought structure (Huang et al., 2023) to divide the generation process into multiple stages, including “problem analysis—code generation—testing verification,” thereby strengthening the model's reasoning pathways. However, these methods generally rely on static generation or closed-loop processes within a single model, making them ill-suited for handling problems with high logical complexity and large test spaces. Moreover, the lack of explicit stage division and collaboration mechanisms results in significant bottlenecks in error detection and functional alignment.

#### 2.1.2 Large language models and code generation

Large Language Models (LLMs) have made groundbreaking progress in code generation tasks, particularly excelling at understanding natural language task descriptions and automatically producing executable code. Representative models such as Codex, CodeLlama, DeepSeek Coder, and StarCoder (Guo et al., 2024; Roziere et al., 2023; Li et al., 2023) are trained and aligned on large-scale code corpora, enabling them to abstract program structures from complex semantic descriptions. These models have demonstrated high accuracy in tasks such as code completion, function implementation, and documentation generation, significantly improving programmers' productivity. However, their capabilities remain limited when faced with complex tasks involving multi-step reasoning, edge-case handling, and algorithm design. In particular, on programming competition datasets, these models often struggle to generate complete solutions that pass all test cases in a single attempt. This reveals their shortcomings in aspects such as contextual planning, state management, and modeling of long-range dependencies. Moreover, the inherent variability in LLM outputs further exacerbates the issue of output instability, posing challenges in safety-critical real-world applications.

#### 2.1.3 Multi-stage prompting and multi-agent approaches

To enhance the performance of large language models (LLMs) on complex code generation tasks, researchers have gradually shifted from single-pass generation to multi-stage reasoning and multi-module collaboration (Jimenez-Romero et al., 2025). At the prompt design level, methods such as Chain-of-Thought (CoT), Self-Planning, and Tree-of-Thought (ToT) guide the model to explicitly output intermediate reasoning steps or subtask plans (Wei et al., 2022; Jiang et al., 2024), thereby improving its capabilities in logical reasoning and task decomposition. From a systems architecture perspective, approaches like Reflexion, Self-Collaboration, and AlphaCodium introduce generation workflows with feedback loops (Shinn et al., 2023; Ridnik et al., 2024), aiming to simulate the iterative loop of coding, testing, and debugging typically employed by human developers. These methods often incorporate virtual roles such as “analyzers” and “testers” to enhance error detection coverage and the specificity of corrections. However, such strategies still face several challenges, including the reliability of test generation, controllability of the feedback mechanism, and the lack of explicit context-sharing among different roles. Therefore, designing a structured code generation framework with clear stage separation, shared context flow, and effective agent collaboration remains a promising and important direction for future research.

### 2.2 Methodology

Our goal is to develop a multi-agent code generation framework tailored for competitive programming problems. The framework simulates the cognitive process of human programmers through four core agents: the Previewing Agent, Blueprint Agent, Coding Agent, and Debugging Agent. Specifically, the Previewing Agent retrieves relevant algorithmic patterns and problem-solving strategies from the models internal knowledge base to establish the technical context. Based on this information, the Blueprint Agent constructs a hierarchical solution plan and selects the optimal strategy through a confidence-based evaluation mechanism. The Coding Agent then implements the solution strictly following competitive programming conventions, while addressing edge cases and complexity constraints. Finally, the Debugging Agent iteratively refines the code using a test-driven feedback mechanism, completing a closed-loop process of analysis, planning, implementation, and verification. To enable effective collaboration among agents, the framework adopts a structured intermediate representation and supports multi-round iterative optimization. This ensures that the final generated solution meets the rigorous standards of competitive programming, while maintaining algorithmic efficiency and robustness.

#### 2.2.1 Previewing Agent

The Previewing Agent is designed to simulate the preliminary understanding and information extraction process that human programmers typically undertake before actual coding. Given a natural language problem description along with example inputs and outputs, the Previewing Agent generates a task summary, suggests potentially relevant algorithm categories, and provides key problem-solving hints to assist subsequent agents in more effectively planning and coding. Unlike traditional retrieval-based approaches, the Previewing Agent does not rely on external knowledge bases; instead, it leverages structured prompts to guide the large language model in performing self-explanation based solely on the problem itself. Our prompt templates are designed to cover task type identification, input/output feature extraction, and strategy association, enabling the model to proactively capture the essential characteristics of the task. Experimental results show that this stage significantly enhances the quality of the subsequent blueprint planning and the accuracy of code generation, particularly for problems involving implicit conditions or edge-case traps.

#### 2.2.2 Blueprint Agent

**Figure 2:**
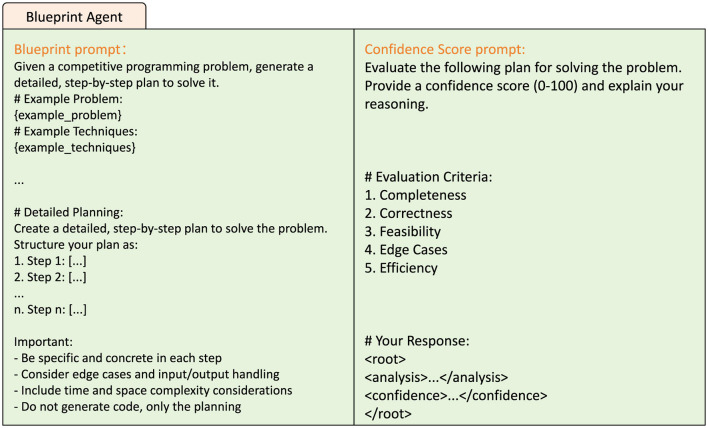
Prompt for Blueprint Agent.

The Blueprint Agent is responsible for generating a structured solution plan for a given problem—essentially, a “design blueprint” for the code. It takes as input the task summary, algorithmic cues, and key points provided by the Previewing Agent, and outputs a step-by-step solution strategy encompassing algorithm logic, edge case handling, and data structure selection. As shown in Figure 2, we designed a structured prompt template that guides the large language model to generate clear problem-solving steps following the sequence of “task objective, key operations, output construction.” Unlike methods such as CoT, the Blueprint Agent produces an implementation-oriented logical flow rather than a generic reasoning trace. To increase the diversity of generated strategies, we set the number of candidate blueprints *k*= 3, producing three distinct solution plans. The system then employs a structured self-evaluation mechanism based on the language model to assess each blueprint. Specifically, an auxiliary prompt is issued asking the model to score the blueprint across five dimensions: completeness, feasibility, edge-case handling, efficiency, and overall quality. Confidence scores are extracted and used to rank the candidate plans, with the highest-scoring blueprint selected as the input for the subsequent coding phase. This structured evaluation mechanism not only improves the rationality of plan selection but also enhances the controllability and interpretability of inter-agent communication. Experimental results demonstrate that high-quality blueprint planning helps the Coding Agent produce more accurate initial code and reduces the number of debugging iterations required.

#### 2.2.3 Coding Agent

The Coding Agent is tasked with generating executable program code based on the step-by-step solution provided by the Blueprint Agent. This stage focuses on accurately mapping the high-level algorithmic plan into concrete implementation details in the target programming language. To ensure consistency and reliability, we constrain the agents input using a structured prompt template that includes the problem description, blueprint plan, and example input-output pairs. Additionally, few-shot exemplars are employed to reinforce stylistic consistency and robust handling of edge cases. After each coding attempt, the agent automatically executes the generated code to verify whether it passes the provided example test cases. If the test is successful, the code is accepted as the final output; otherwise, control is handed over to the Debugging Agent for refinement. This automatic verification mechanism ensures functional correctness while avoiding redundant or ineffective generations.

#### 2.2.4 Debugging Agent

The Debugging Agent is designed to simulate the behavior of human programmers in repairing code based on test feedback. When the code generated by the Coding Agent fails to pass all example test cases, the Debugging Agent performs step-by-step analysis and revision by leveraging the original problem description, the blueprint plan, and the faulty code. We guide the large language model to follow a structured reasoning pattern of “error analysis, revision strategy, code repair,” and explicitly require it to first complete any missing logical steps before applying modifications, thereby ensuring consistency and controllability throughout the repair process. Unlike approaches such as Reflexion (Shinn et al., 2023), which rely on generating additional test cases, our method exclusively uses the original example I/O, avoiding potential inaccuracies introduced by synthetic samples. To limit unnecessary repair cycles, we set the maximum number of debugging iterations to *t* = 5; after five unsuccessful attempts, the system automatically reverts to the next-best blueprint for a fresh attempt. Experimental results show that the Debugging Agent significantly improves the overall code generation success rate, making it one of the most critical components of the framework.

## 3 Experiments

### 3.1 Datasets

To comprehensively evaluate the performance of Blueprint2Code across varying levels of task complexity, we conducted experiments on five datasets: two widely used public benchmarks, HumanEval and MBPP; their extended versions, HumanEval-ET and MBPP-ET; and the competitive programming dataset APPS sourced from a public platform. HumanEval primarily consists of function-level implementation tasks, emphasizing the models ability to understand natural language descriptions and align with test cases. MBPP focuses on assessing fundamental programming skills. HumanEval-ET and MBPP-ET enhance the coverage and complexity of test cases, enabling more fine-grained evaluation of model robustness. The APPS dataset includes real-world programming problems ranging from basic to competition-level difficulty, featuring open-ended tasks with rich input-output examples and more natural language descriptions. It serves as a key benchmark for assessing the comprehensive capabilities of large language models.

### 3.2 Baselines

We compared Blueprint2Code against several baseline and state-of-the-art approaches. Direct generation refers to prompting LLMs to write code based solely on the dataset-provided problem descriptions, relying entirely on the models internal capabilities. Chain-of-Thought (CoT) encourages the model to reason step-by-step before producing a solution. Self-Planning decomposes the task into separate planning and execution stages. Reflexion enhances code accuracy by prompting the model to recall relevant techniques and algorithms from prior training data.

### 3.3 Related settings

We utilized OpenAI's large language models, including ChatGPT (based on GPT-3.5-turbo) and GPT-4 (based on GPT-4o), for all experiments. The evaluation metric adopted was Pass@k, where a problem is considered solved if at least one of the k enerated code samples passes all test cases. In all experiments, we fixed the number of blueprint candidates *k* = 3 and the maximum number of debugging iterations *t*= 5. These parameters were selected based on commonly adopted settings in related work and preliminary empirical analysis, balancing solution diversity, system stability, and computational cost. While the current configuration demonstrated consistent performance across various tasks and model settings, future work will further explore the trade-off between blueprint diversity and debugging overhead by varying *k* and *t*, aiming to systematically optimize both generation quality and efficiency.

## 4 Results

To comprehensively evaluate the performance of Blueprint2Code on code generation tasks, we compared it against several representative methods across five datasets, including Direct, Chain-of-Thought (CoT), Self-Planning, Reflexion, and MapCoder. All methods were tested under the same evaluation protocol and deployed on two large language model platforms: ChatGPT (based on GPT-3.5-turbo) and GPT-4o (gpt-4o). In addition, to further assess the applicability of our framework in resource-constrained environments, we introduced GPT-4o-mini (8B) as a lightweight model and conducted a comparative analysis to evaluate the transferability and performance stability of Blueprint2Code under smaller model settings.

Performance across problem types: although our evaluation focuses on aggregate pass@1 scores, the datasets themselves cover diverse problem categories. HumanEval primarily consists of functional programming tasks and algorithmic puzzles, while MBPP emphasizes basic algorithmic and data structure exercises. HumanEval-ET and MBPP-ET introduce more challenging boundary cases, and APPS covers a wide spectrum from beginner-level programming to competitive-level problems requiring multi-step reasoning. Based on qualitative inspection, Blueprint2Code tends to achieve the largest relative gains in tasks that require explicit boundary handling (common in MBPP-ET) and multi-stage solution planning (frequent in APPS). For simpler algorithmic categories (e.g., straightforward string manipulation in MBPP), the performance gap to baselines is smaller, indicating that our multi-agent coordination provides the most benefit in complex reasoning scenarios.

### 4.1 Comparative experiments with large models

Table 1 presents the Pass@1 accuracy of different methods on five benchmark datasets—HumanEval, MBPP, HumanEval-ET, MBPP-ET, and APPS—using two language models: GPT-3.5-turbo and GPT-4o. Overall, Blueprint2Code consistently achieves the best performance across all datasets and model configurations, demonstrating strong task adaptability and model generalization capabilities.

**Table 1:** Pass@1 accuracy (%) of different methods across five code generation benchmarks using GPT-3.5-turbo and GPT-4o.

**LLM**	**Approach**	**HumanEval**	**MBPP**	**HumanEval-ET**	**MBPP-ET**	**APPS**
GPT-3.5-turbo	Direct	48.2	49.8	43.7	36.8	6.0
	CoT	68.4	54.5	54.2	40.1	7.3
	Self-Planning	60.3	55.7	46.2	42.5	10.6
	Reflexion	67.1	73.0	52.4	48.1	–
	MapCoder	80.5	78.4	70.1	54.4	11.3
	Blueprint2Code	**90.8** (+12.8)	**85.4** (+8.9)	**75.6** (+7.8)	**57.2** (+5.1)	**12.0** (+5.8)
GPT-4o	Direct	80.1	81.1	73.8	55.6	12.7
	CoT	89.0	82.4	61.6	56.2	11.3
	Self-Planning	85.4	75.8	62.2	52.1	14.7
	Reflexion	91.0	78.3	78.7	51.9	–
	MapCoder	93.9	83.1	82.9	57.7	22.0
	Blueprint2Code	**96.3** (+2.6)	**88.4** (+6.4)	**86.5** (+4.3)	**59.4** (+2.9)	**24.6** (+11.8)

Relative improvements over MapCoder are shown in parentheses. The bold values indicate the best results achieved on the corresponding dataset.

Under the GPT-3.5-turbo setting, Blueprint2Code achieves Pass@1 scores of 90.8%, 85.4%, 75.6%, 57.2%, and 12.0% on HumanEval, MBPP, HumanEval-ET, MBPP-ET, and APPS, respectively. Compared with the Direct baseline, Blueprint2Code demonstrates significantly improved performance across all datasets, particularly on tasks requiring complex reasoning and robust generalization. Moreover, when compared to the structure-aware MapCoder framework, it achieves relative improvements of 12.8%, 8.9%, 7.8%, 5.1%, and 5.8%, respectively. These results confirm the effectiveness of the proposed multi-agent design in enhancing code synthesis quality and reliability under diverse task conditions.

Under the GPT-4o model configuration, Blueprint2Code further improves its performance, achieving Pass@1 scores of 96.3%, 88.4%, 86.5%, 59.4%, and 24.6% on HumanEval, MBPP, HumanEval-ET, MBPP-ET, and APPS, respectively. Compared with the Direct baseline, Blueprint2Code demonstrates consistently superior performance across all benchmarks. In comparison with the structure-aware MapCoder framework, it achieves relative improvements ranging from 2.6% to 11.8%, indicating its enhanced effectiveness in structured reasoning, test case coverage, and robust generalization on complex programming tasks.

Taken together, these results demonstrate that Blueprint2Code exhibits stable performance advantages under both medium-capacity and high-capacity models. In particular, its continued strong performance on the extended benchmarks HumanEval-ET and MBPP-ET—designed to include broader edge case coverage—further validates its generalization ability and adaptability to complex task scenarios.

### 4.2 Transferability experiments on lightweight models

**Figure 3:**
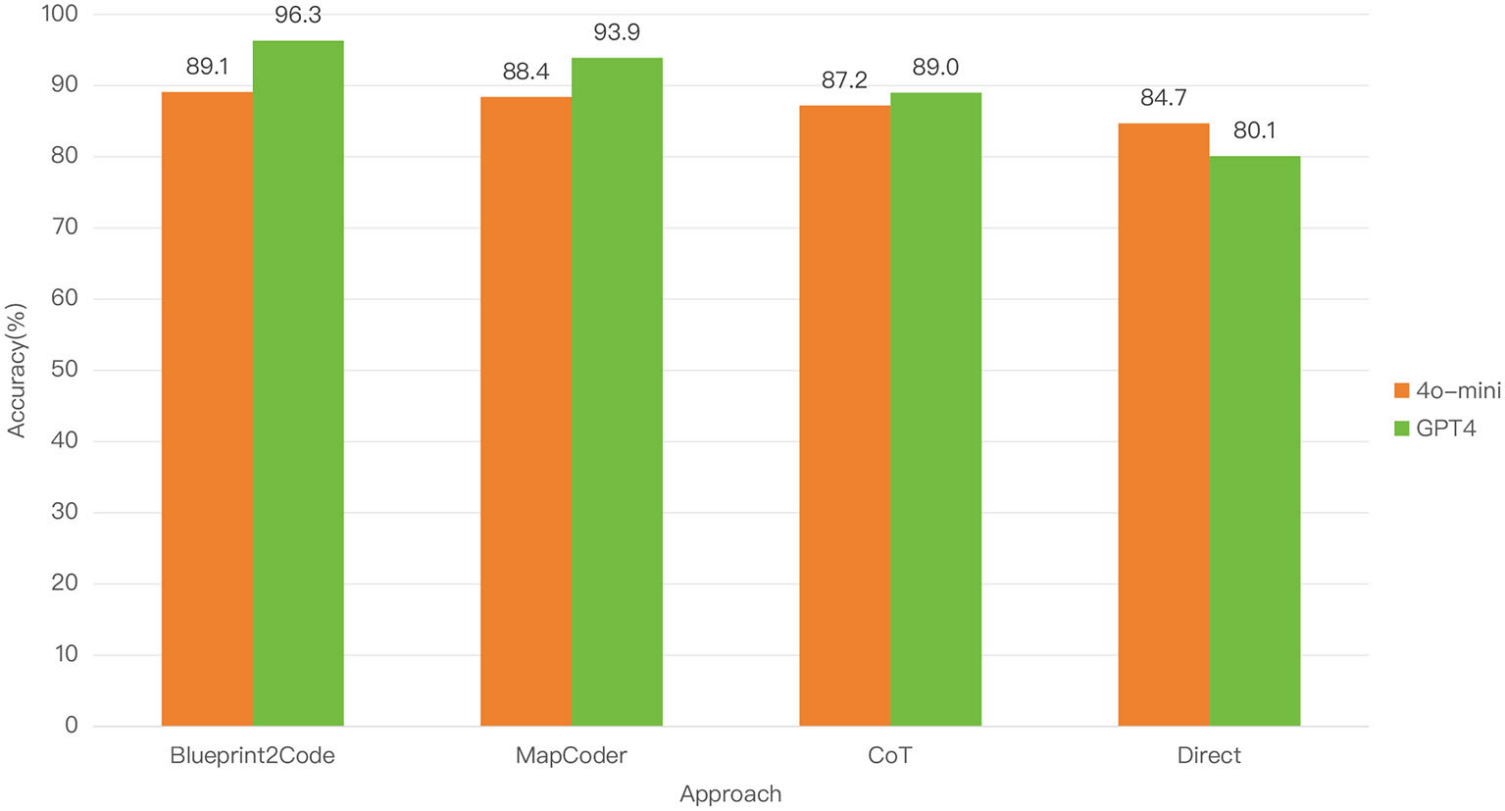
Performance of Blueprint2Code on lightweight models.

To further assess the lightweight applicability of Blueprint2Code and its performance in resource-constrained environments, we conducted additional experiments on the HumanEval dataset using GPT-4o-mini (8B), and compared it against MapCoder and other baseline methods. As shown in Figure 3, although most methods (except for Direct) experienced slight performance degradation on GPT-4o-mini compared to their results on GPT-4o, Blueprint2Code maintained a clear performance advantage over all baselines. This demonstrates that Blueprint2Code remains effective even under small model settings, with strong task decomposition and coordination capabilities, indicating good transferability and practical utility. These results further validate the complementary role of the multi-agent architecture in enhancing model performance—especially for LLMs with weaker reasoning abilities, where inter-agent collaboration helps compensate for limitations in planning and debugging. It is worth noting that although the current experiments rely on commercial models provided by OpenAI, the design of Blueprint2Code is model-agnostic, with modular interfaces that can be easily extended to local open-source models (e.g., DeepSeek Coder, CodeGeeX2) or integrated with lightweight inference optimization strategies (Guo et al., 2024; Bi et al., 2024; Zheng et al., 2023), paving the way for deployment on edge devices or in low-resource scenarios. Additionally, automatically adaptive collaboration strategies among agents—such as dynamically adjusting the execution sequence and number of agents based on task complexity—represent promising directions for future research.

### 4.3 Ablation study

To analyze the contribution of each agent in the Blueprint2Code framework, we conducted an ablation study on the HumanEval dataset by selectively removing the Previewing Agent, Blueprint Agent, and Debugging Agent, respectively, under the GPT-3.5-turbo setting. The results are shown in Table 2. The experiments reveal that removing any of the agents leads to performance degradation, though the extent varies. Specifically, removing the Debugging Agent results in the most significant drop, with accuracy falling to 64.6%—a decrease of 28.9% compared to the full system. This highlights the critical role of debugging in improving code correctness and test case pass rate. Removing the Blueprint Agent and Preview Agent results in accuracies of 77.4 and 76.8%, reflecting decreases of 14.8 and 15.4%, respectively, suggesting that structured planning and task comprehension also play essential roles in ensuring code quality. These findings demonstrate that the multi-agent architecture achieves its effectiveness through the synergy of all its components. In complex code generation tasks, early-stage task understanding and structured planning provide a solid foundation for high-quality initial code, while the debugging phase serves as a vital mechanism for correctness assurance. In future work, we plan to explore dynamic agent adaptation strategies, such as automatically adjusting the number of stages or iteration rounds based on task complexity, to further improve the framework's efficiency and stability.

**Table 2:** Ablation results of Blueprint2Code framework.

**Previewing Agent**	**Blueprint Agent**	**Debugging Agent**	**Pass@1 (%)**	**Performance drop**
×	✓	✓	76.8	−15.4
✓	×	✓	77.4	−14.8
✓	✓	×	64.6	−28.9

✓ indicates the agent is included, × indicates it is removed.

### 4.4 Robustness and error analysis

Robustness across models and ablations: the proposed Blueprint2Code consistently outperforms all baselines across three different model configurations (GPT-3.5-turbo, GPT-4o, and GPT-4o-mini), indicating that its improvements are not tied to a specific model capacity. The ablation study (Table 2) further demonstrates that removing any agent leads to substantial performance drops (—14.8% to —28.9% on HumanEval), confirming the robustness of the multi-agent design.

Beyond agent removal: in addition to the “all-agents removed” baseline, we evaluated partial configurations (e.g., removing the Blueprint Agent while keeping Debug Agent), and the results confirm that each component contributes positively to the overall performance. Future work will explore dynamic agent scheduling strategies based on problem complexity to further enhance efficiency and robustness.

## 5 Conclusion and future works

This paper presents Blueprint2Code, a multi-agent code generation framework designed for complex programming tasks. Inspired by the cognitive workflow of human developers, the generation process is divided into four distinct stages: previewing, blueprint planning, code implementation, and debugging—each handled by a dedicated agent. Through structured prompting and modular design, the framework enhances the capabilities of large language models in task understanding, strategic planning, and error correction. To validate its effectiveness, we conducted comprehensive evaluations on five datasets: two widely used public benchmarks (HumanEval and MBPP), their extended versions (HumanEval-ET and MBPP-ET), and the APPS competitive programming dataset. Experimental results show that Blueprint2Code consistently outperforms existing methods such as CoT, Reflexion, and MapCoder in terms of Pass@1 accuracy. Notably, it maintains strong performance even under resource-constrained settings with smaller models, demonstrating excellent generality and practical value. Overall, by introducing an explicit multi-stage collaboration mechanism and agent specialization, Blueprint2Code improves the stability, interpretability, and scalability of code generation systems, offering a novel approach to building reliable and generalizable automated programming frameworks.

Despite the strong performance of Blueprint2Code, there remain several promising directions for future research. First, the current agent collaboration follows a fixed execution order; future work may explore more flexible strategies, such as dynamically adjusting the sequence and frequency of agent invocation based on task complexity. Second, adapting the framework to local open-source models—such as DeepSeek Coder and CodeGeeX2—represents an important step toward real-world deployment. Finally, we plan to extend Blueprint2Codes capabilities to support multilingual and multitask scenarios, such as debugging document generation and cross-language translation, further enhancing its practicality and extensibility.

## Data Availability

Publicly available datasets were analyzed in this study. This data can be found at: https://github.com/openai/human-eval, https://github.com/google-research/google-research/tree/master/mbpp, and https://github.com/hendrycks/apps.
